# Dysregulation of glycogen metabolism in cardiomyopathy caused by glycogenin-1 (*GYG1*) missense variants

**DOI:** 10.1210/clinem/dgag212

**Published:** 2026-05-21

**Authors:** Kittichate Visuttijai, Carola Hedberg-Oldfors, Oscar Braun, Elisabet Englund, Emma Glamuzina, Clinton Turner, Kristina Vukusic, Joakim Sandstedt, Göran Dellgren, Kristjan Karason, Anders Oldfors

**Affiliations:** Department of Laboratory Medicine, Institute of Biomedicine, University of Gothenburg, 413 90 Gothenburg, Sweden; Department of Clinical Pathology, Sahlgrenska University Hospital, Region Västra Götaland, 413 45 Gothenburg, Sweden; Department of Laboratory Medicine, Institute of Biomedicine, University of Gothenburg, 413 90 Gothenburg, Sweden; Department of Clinical Pathology, Sahlgrenska University Hospital, Region Västra Götaland, 413 45 Gothenburg, Sweden; Department of Clinical Genetics and Genomics, Sahlgrenska University Hospital, Region Västra Götaland, 413 45 Gothenburg, Sweden; Cardiology, Department of Clinical Sciences, Lund University and Skåne University Hospital, 221 85 Lund, Sweden; Pathology, Department of Clinical Sciences, Lund University and Skåne University Hospital, 221 85 Lund, Sweden; Adult and Paediatric National Metabolic Service, Starship Children's Health, Te Whatu Ora Health New Zealand, Auckland 1023, New Zealand; Anatomical Pathology, Pathology and Laboratory Medicine, Auckland City Hospital, Te Whatu Ora Health New Zealand, Auckland 1060, New Zealand; Department of Laboratory Medicine, Institute of Biomedicine, University of Gothenburg, 413 90 Gothenburg, Sweden; Department of Clinical Chemistry, Sahlgrenska University Hospital, Region Västra Götaland, 413 45 Gothenburg, Sweden; Department of Laboratory Medicine, Institute of Biomedicine, University of Gothenburg, 413 90 Gothenburg, Sweden; Department of Clinical Chemistry, Sahlgrenska University Hospital, Region Västra Götaland, 413 45 Gothenburg, Sweden; Department of Cardiothoracic Surgery, Sahlgrenska University Hospital, Region Västra Götaland, 413 45 Gothenburg, Sweden; Department of Cardiology, Sahlgrenska University Hospital, Region Västra Götaland, 413 45 Gothenburg, Sweden; Department of Laboratory Medicine, Institute of Biomedicine, University of Gothenburg, 413 90 Gothenburg, Sweden; Department of Clinical Pathology, Sahlgrenska University Hospital, Region Västra Götaland, 413 45 Gothenburg, Sweden

**Keywords:** *GYG1*, glycogen storage disease XV, polyglucosan body myopathy 2, PGBM2, cardiomyopathy, proteomic profiling

## Abstract

**Context:**

Glycogen storage diseases are inherited disorders of glycogen metabolism and are commonly associated with hypoglycemia, hepatic dysfunction, or skeletal and cardiac myopathy, depending on the affected enzyme. Glycogen storage disease type 15 (GSD15) is caused by pathogenic variants in *GYG1*, which encodes glycogenin-1, the autoglucosylating primer required for glycogen synthesis. GSD15 is characterized by cardiomyopathy and storage of abnormal glycogen and polyglucosan in cardiomyocytes.

**Objective:**

To understand the pathobiology of GSD15, we investigated the storage material in heart explants from 2 previously reported patients and described a new case of GSD15.

**Methods:**

The characteristic storage material was investigated using laser capture microdissection followed by quantitative mass spectrometry (MS) and immunohistochemistry comparing differentially abundant proteins in the storage material with normal-appearing cytoplasmic regions from the same patient. Global protein dysregulation in GSD15 was assessed by quantitative MS of whole-myocardial tissue samples from patients and normal controls.

**Results:**

The storage material was enriched in proteins involved in glycogen metabolism, including glycogen synthase, UDP-glucose pyrophosphorylase 2, glycogenin-1, glycogen phosphorylase, and glycogen debranching enzyme. Sequestosome 1 (p62) and desmin were also enriched, without evidence of increased autophagocytosis. Whole-tissue analyses revealed upregulation of cardiomyopathy-associated biomarkers and downregulation of mitochondrial proteins, suggesting impaired energy metabolism contributing to congestive heart failure.

**Conclusion:**

In GSD15, storage of abnormal glycogen in cardiomyocytes is associated with enrichment of specific proteins involved in glycogen metabolism, contributing to dysregulation of glycogen turnover. This dysregulation results in polyglucosan accumulation, disruption of sarcomeric and mitochondrial architecture, and progressive fibrosis. No disease-modifying therapy currently exists for GSD15; future strategies based on substrate reduction, enhancement of autophagy, or gene therapy require further investigation.

In recent decades, advances in biochemistry and molecular genetics have transformed the understanding of inborn errors of metabolism and enabled many patients, children and adults, to receive effective treatments such as gene therapy and enzyme replacement therapy. In this context, glycogen and its metabolism play a central role in glucose homeostasis, energy metabolism, and adenosine triphosphate production in multiple tissues, including muscle, liver, and heart (1). Disruptions in glycogen metabolism give rise to a heterogeneous group of disorders, collectively known as glycogen storage diseases (GSDs). These disorders are caused by defects in enzymes or regulatory proteins involved in glycogen synthesis or degradation, leading to impaired glycogen homeostasis. As a result, glycogen may accumulate abnormally, be insufficiently produced, or fail to be properly mobilized into glucose. Because glycogen is essential for maintaining systemic glucose balance and meeting energy demands, GSDs primarily affect tissues with high glycogen turnover, particularly the liver and skeletal muscle.

Hepatic forms of GSD typically present with fasting hypoglycemia, hepatomegaly, and metabolic derangements, whereas muscle forms are characterized by exercise intolerance, recurrent rhabdomyolysis, myalgia, and/or progressive muscle weakness and atrophy. Only a few GSDs affect the heart muscle, including GSD0 (*GYS1*, muscle glycogen synthase deficiency), GSD2 (*GAA*, Pompe disease, α glucosidase deficiency), GSD3 (*AGL*, debranching enzyme deficiency), GSD4 (*GBE1*, α 1,4-glucan branching enzyme deficiency), and GSD15 (*GYG1*, glycogenin-1 deficiency).

Glycogenin-1 is considered a key protein for de novo glycogen synthesis and is therefore of central importance for regulation of glycogen and glucose metabolism (2). Glycogenin-1 is a glycosyltransferase that initiates glycogen formation by catalyzing 2 autoglucosylation reactions using UDP-glucose as the sugar donor. In the first step, a glucose moiety is covalently attached to the hydroxyl group of tyrosine 195 via an O-glycosidic bond (3-5). This priming event is followed by the extension of the nascent chain through successive α-1,4-glucosidic linkages, generating an oligosaccharide comprising approximately 8 to 13 glucose residues (6, 7). The oligosaccharide is subsequently elongated by glycogen synthase, while α-1,6 branching points are introduced by the branching enzyme, culminating in the formation of mature glycogen (1, 8, 9) (Fig. 1). Glycogenin-1, encoded by *GYG1*, is ubiquitously expressed, but in humans, a second isoform is present, glycogenin-2 (*GYG2*), which is predominantly expressed in the liver, with minimal or no expression in skeletal muscle and heart (10, 11).

**Figure 1:**
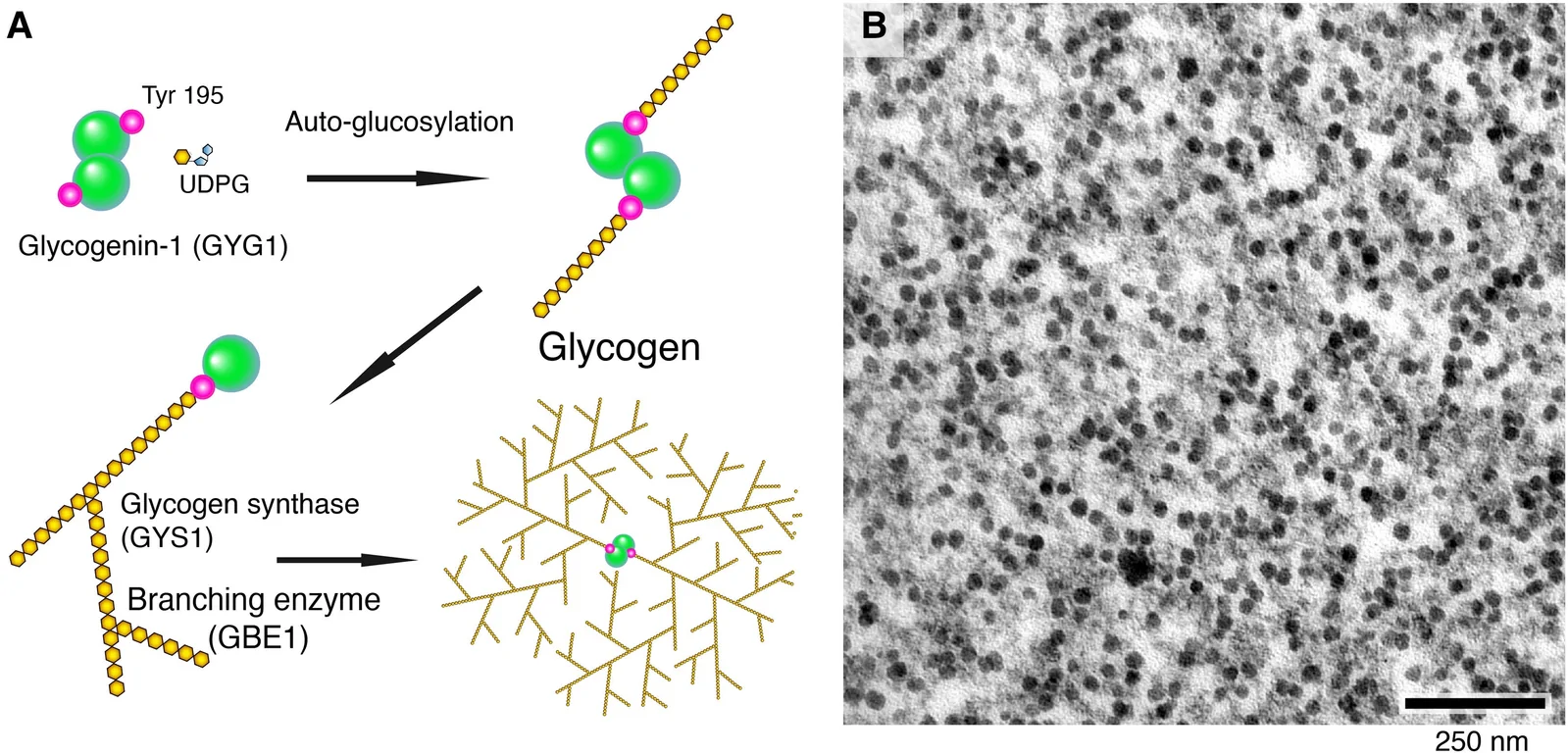
Glycogen de novo synthesis. A, Glycogenin-1 initiates glycogen synthesis by catalyzing 2 self-glucosylation reactions using UDP-glucose (UDPG) as the glucose donor. First, a glucose residue is covalently attached to tyrosine 195 through an O-glycosidic bond, followed by the formation of several α-1,4-glucosidic linkages that extend the chain to an oligosaccharide of roughly 8 to 13 glucose units. This primer serves as a substrate for glycogen synthase, which further elongates the chain, while branching enzyme introduces α-1,6 linkages to generate soluble glycogen particles. B, Each glycogen granule, illustrated in the electron micrograph from muscle tissue, has an approximate diameter of 20 nm.

Diseases associated with genetic variants in *GYG1* have been reported with 2 different clinical presentations, with only partial overlap. One phenotype presents as a cardiomyopathy and is referred to as glycogen storage disease type 15 (GSD15; OMIM No. 613507), and the other as a pure skeletal myopathy called polyglucosan body myopathy 2 (PGBM2; OMIM No. 616199).

GSD15, the first disease described to be associated with glycogenin-1 dysfunction, was reported in 2010 (12). The patient suffered from arrhythmias and progressive cardiomyopathy with large inclusions of abnormal glycogen in the cardiomyocytes. Later, 3 additional cases with a similar glycogen storage cardiomyopathy and dysfunctional glycogenin-1 were reported (13). Two of them had a severe dilated cardiomyopathy and underwent cardiac transplantation.

PGBM2 seems to be more common than GSD15 and is not associated with cardiomyopathy. This entity was first described in 2014 in 7 patients (14), but at present, more than 30 patients have been reported. PGBM2 usually presents at middle age or later with progressive muscle weakness (14-17). Muscle histopathology is characterized by storage of abnormal glycogen partly with features of polyglucosan in the muscle fibers (14). PGBM2 is usually associated with biallelic truncating *GYG1* mutations, and no or very low levels of glycogenin-1 are present in the muscle tissue.

This study was undertaken to shed further light on the pathobiology and clinical phenotype of the cardiomyopathy, which is characteristic of GSD15. We performed proteomic and immunohistochemical (IHC) analyses of the storage material in the heart and follow-up of 2 previously reported case patients, who eventually underwent cardiac transplantation, and also describe a new patient with GSD15.

## Materials and methods

This study was conducted in accordance with the Declaration of Helsinki and was approved by the Swedish Ethical Review Authority. Informed consent was obtained from the investigated individuals. The medical history of 3 patients is reported in the Results section (2 on follow-up from previous publications and 1 newly identified).

### Experimental design

A schematic overview of the materials and methods for each patient used in this paper is shown in Fig. 2A to 2C.

**Figure 2:**
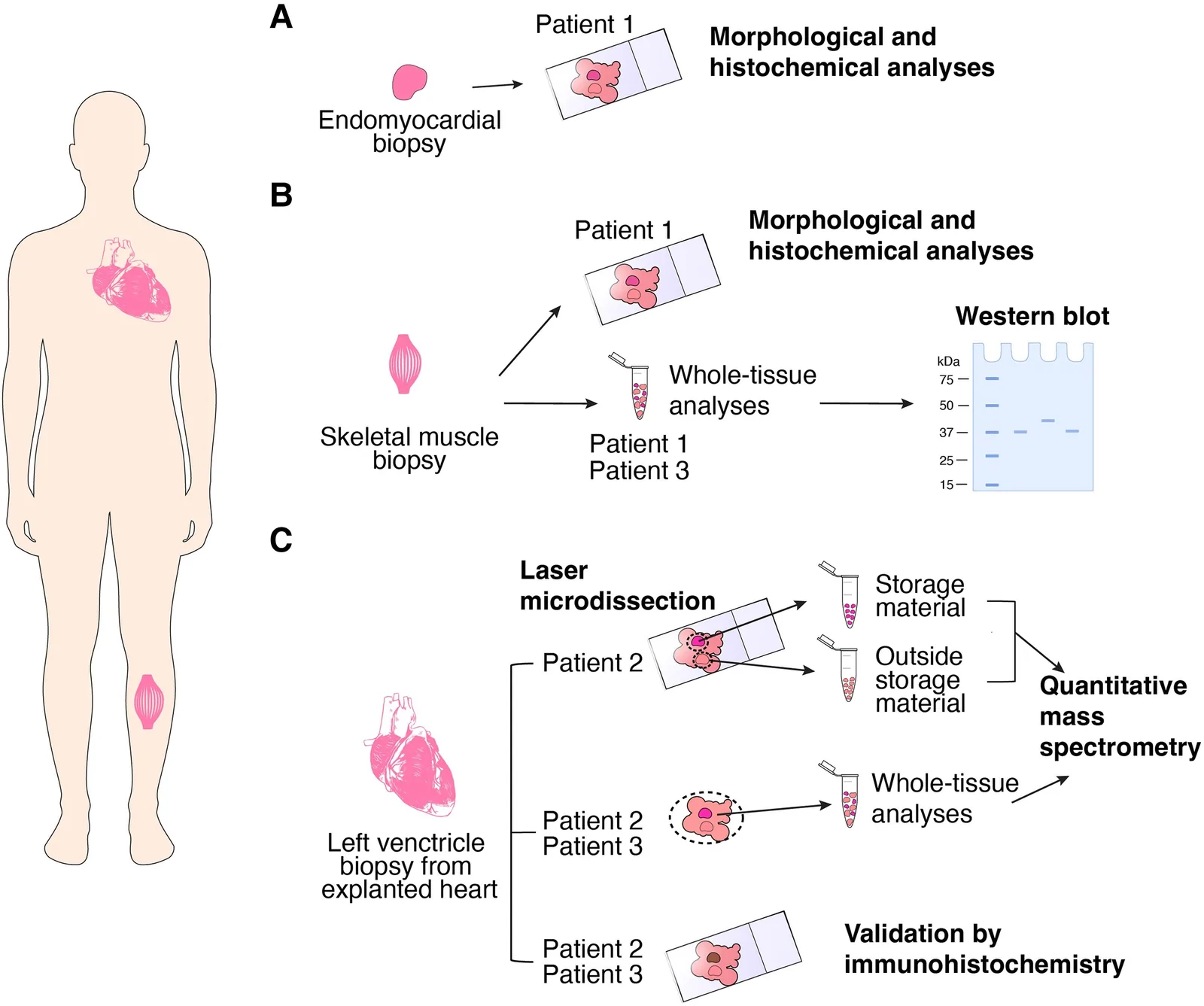
Overview of tissue samples and analytical approaches. A, A heart biopsy from patient 1 was used for morphological and histochemical analyses. B, Skeletal muscle biopsies were used for morphological and histochemical analyses (patient 1) and for Western blot analysis (patients 1 and 3). C, Heart explants from patients 2 and 3 were used for proteomic analyses and immunohistochemistry (IHC). To identify proteins enriched in PAS-positive inclusions containing storage material, laser capture microdissection was performed on heart explants from patient 2, isolating 50 000 µm^2^ of inclusion-containing regions and 50 000 µm^2^ of cytoplasmic regions outside the inclusions, followed by LC-MS^3^ analysis. For whole-tissue proteomic analysis by LC-MS^3^, left ventricular specimens (5-10 mm in diameter) from explanted hearts of patients 2 and 3 and four control hearts were fresh-frozen, cryosectioned, and processed for protein extraction (1 mg per sample, in triplicate). IHC was performed on cryosections from the same specimens.

### Morphological and histochemical analyses

In patient 1, an endomyocardial biopsy at age 41 years was fixed in formalin and embedded in paraffin. Routine staining of the sections included hematoxylin and eosin for overall histopathological evaluation and periodic acid–Schiff reagent (PAS) for glycogen (Fig. 2A). A skeletal muscle biopsy from the vastus lateralis muscle of the quadriceps muscle of patient 1 was fresh-frozen and cryostat sections were processed for morphological analyses according to routine methods (18) (Fig. 2B). From the explanted hearts of patients 2 and 3, aged 48 and 36 years, respectively, left ventricular specimens were fresh-frozen in liquid nitrogen and used for histology and IHC (Fig. 2C). Histochemical and IHC analyses were performed on 6-µm-thick sections using standard protocols (18), with immunostaining carried out on a Dako Autostainer and EnVision FLEX High pH kit in accordance with the manufacturer's standard protocol, which was described previously (19). Primary antibodies and dilutions are listed in Table 1. The selection of antibodies was based on results from proteomic analysis of the storage material (discussed later) and our previous studies on polyglucosan inclusions in skeletal muscle in glycogenin-1 deficiency (19) and RBCK1 deficiency (20). For analysis of the mitochondrial membrane protein VDAC1 and respiratory chain complexes I, II, and IV, a quadruple immunofluorescence method was applied (21). Primary and secondary antibodies and dilutions are listed in Table 1. Electron microscopy was performed on endomyocardial biopsy specimens from patients 2 and 3 using standard techniques (18).

**Table 1:** Primary and secondary antibodies used for immunohistochemistry and immunofluorescence

Antibody (gene symbol/ isotype/conjugation)	Clone	Host	Supplier (product code, RRID)	Dilution
**Primary antibodies for immunohistochemistry**				
Debranching enzyme (*AGL*)		Rabbit (pAb)	Abgent (AP2402B, RRID: AB_2305340)	1:200
Desmin (*DES*)	D33	Mouse (mAb)	Dako (M0760, RRID: AB_2335684)	1:40
Glycogen phosphorylase, brain (*PYGB*)		Rabbit (pAb)	Proteintech (12075-1-AP, RRID: AB_2174885)	1:400
Glycogen synthase (*GYS1*)		Rabbit (pAb)	Atlas Antibodies (HPA041598, RRID: AB_2677571)	1:1000
Glycogenin-1 (*GYG1*)	E-11	Mouse (mAb)	Santa Cruz (sc-271109, RRID: AB_10610491)	1:1000
Laforin (*EPM2A*)	N84/37	Mouse (mAb)	Millipore (MABN606, RRID: AB_3750359)	1:250
LAMP2 (*LAMP2*)	H4B4	Mouse (mAb)	Abcam (ab25631, RRID: AB_470709)	1:200
LC3 (*MAP1LC3B*)		Rabbit (pAb)	Sigma-Aldrich (L7543, RRID: AB_796155)	1:1000
Phosphofructokinase (*PFKM*)	EPR10734(B)	Rabbit (mAb)	Abcam (ab154804, RRID: AB_3076356)	1:2000
Sequestosome 1/p62 (*SQSTM1)*	D-3	Mouse (mAb)	Santa Cruz (sc-28359, RRID: AB_628279)	1:2000
Ubiquitin (*UBB*)	P4D1-A11	Mouse (mAb)	Sigma-Aldrich (05-944, RRID: AB_441944)	1:100
**Primary antibodies for immunofluorescence**				
Complex I (*NDUFB8*, IgG1)	20E9DH10C12	Mouse (mAb)	Abcam (ab110242, RRID: AB_10859122)	1:200
Complex II (*SDHB*, IgG2a)	21A11AE7	Mouse (mAb)	Abcam (ab14714, RRID: AB_301432)	1:200
Complex IV (*MTCO1*, IgG2a)	1D6E1A8	Mouse (mAb)	Abcam (ab14705, RRID: AB_2084810)	1:200
Porin (*VDAC1*, IgG2b)	20B12AF2	Mouse (mAb)	Abcam (ab14734, RRID: AB_443084)	1:100
Heparan Sulfate Proteoglycan/Perlecan (*HSPG2*, IgG2aκ)	A7L6	Rat (mAb)	Merck (MAB1948P, RRID: AB_10615958)	1:1000
**Secondary antibodies for immunofluorescence**				
Mouse IgG1 (Alexa Fluor 488)		Goat	ThermoFisher Scientific (A-21121, RRID: AB_2535764)	1:200
Mouse IgG2a (Alexa Fluor 647)		Goat	ThermoFisher Scientific (A-21241, RRID: AB_2535810)	1:100
Mouse IgG2b (Brilliant Violet 421)		Goat	Jackson ImmunoResearch (115-675-207, RRID: AB_2651090)	1:100
Rat IgG (H + L) (Alexa Fluor 568)		Goat	ThermoFisher Scientific (A-11077, RRID: AB_2534121)	1:250

Abbreviations: IgG, immunoglobulin G; LAMP2, lysosomal-associated membrane protein 2; LC3, microtubule-associated protein 1A/1B-light chain 3; mAb, monoclonal antibody; pAb, polyclonal antibody; RRID, research resource identifier.

### Western blot analysis

Western blot analysis was performed on proteins extracted from fresh-frozen muscle specimens of patients 1 and 3 (see Fig. 2B). For glycogenin-1 detection, cryosections were pretreated with α-amylase at 37 °C for an hour before extraction in sodium dodecyl sulfate–urea buffer. A total of 5 µg of protein were separated on NuPAGE 4% to 12% Bis-Tris gels (Thermo Scientific) and transferred to poly(vinylidene fluoride) membranes. Membranes were incubated overnight at 4 °C with primary antibody, anti-GYG1 (1:500, clone 3B5, H00002992-M07, Abnova, RRID: AB_530073), followed by horseradish peroxidase–conjugated secondary antibody (1:1000, 32430, Thermo Scientific, RRID: AB_1185566) and detection with SuperSignal West Femto (Thermo Scientific) on a Fujifilm LAS-4000 chemiluminescence system. Coomassie staining (SimplyBlue SafeStain, Invitrogen) of myosin heavy chain was used as a loading control.

### Laser capture microdissection and quantitative mass spectrometry

For laser capture microdissection (LCM) of storage material, fresh-frozen myocardial specimens from the explanted heart of patient 2 were cryosectioned at 10 µm and mounted on polyethylene naphthalate MembraneSlide 1.0 PENs (415190-9051-000, Zeiss) and stained using a modified PAS protocol (20). Microdissection (PALM, Zeiss) was performed to isolate regions containing either PAS-positive storage material or cardiomyocyte cytoplasm outside the storage material, in total 50 000 µm^2^ from each region, and collected in AdhesiveCap 500 Opaque (415190-9201-000, Zeiss) (Fig. 2C). Triplicate samples were prepared for both types of regions to identify proteins that were enriched in the storage material in the cardiomyocytes.

For whole-tissue analyses, specimens, 5 to 10 mm in diameter, from the left ventricle (LV) of explanted hearts from patients 2 and 3 and four controls (discussed later) were fresh-frozen. Cryosections were prepared for protein extraction (1 mg each, in triplicate).

Proteomic profiling was conducted by quantitative mass spectrometry (MS) on both laser-microdissected regions and whole-tissue homogenates (Fig. 2C). The protein extracts were digested with trypsin, and peptides were labeled with tandem mass tags (TMTs) for protein quantification. Detailed procedures of Nano LC*-*MS^3^ for microdissected specimens and whole-tissue homogenates are provided in the Supplementary Material File (22).

Protein data were partly analyzed with the software Omics Playground (BigOmicsAnalytics, v3.5.24) (23). To identify differentially expressed proteins, the data were log2-transformed, and then, for each protein, log2 fold change (log2FC) and *P* values were computed using the Welch *t* test for PAS-positive storage material vs cytoplasm outside storage material as well as whole-tissue from patients vs controls. To control for multiple comparisons, the Benjamini-Hochberg method was used to adjust the *P* values, and proteins with a false discovery rate less than .05 were considered statistically significant.

Pathway analysis and protein-protein interaction analysis were performed by applying different search tools and web resources such as Proteomaps website (https://www.proteomaps.net/ (24)) and STRING website (https://www.string-db.org/ (25)).

### Controls

Control samples for whole-tissue MS and for IHC were obtained from the LV of donor hearts from 4 men, mean age 65 years (range, 54-74 years). These hearts were considered normal but, for various reasons, not suitable for transplantation and saved for research purposes. Specimens measuring 5 to 10 mm in diameter were fresh-frozen in liquid nitrogen and stored at −80 °C. Cryosections were stained with hematoxylin and eosin to ascertain that the material was composed of representative myocardium with normal histology.

## Results

### Patient case reports

Clinical characteristics of the patients are summarized in Table 2.

**Table 2:** Clinical characteristics of the 3 patients with GSD15

	Patient 1	Patient 2	Patient 3
Sex	Male	Male	Male
Age at first cardiac presentation, year	36	36	27
Initial clinical signs	Recent onset of fatigue; at age 41 y, sustained ventricular tachycardia with hemodynamic instability	Palpitations and exertional chest pain	Out-of-hospital cardiac arrest due to ventricular fibrillation, successfully resuscitated
Initial cardiac imaging (CMR)	Age 41 y: mildly reduced EF extensive LGE especially in lateral wall and basal septum	Age 45 y: severely dilated LV with marked dysfunction and extensive mid-myocardial/subepicardial LGE	Age 27 y: extensive LGE involving inferior and septal LV walls and dilated LV with EF 40%
Coronary angiography	Normal	Normal	Normal
Myocardial biopsy	Extensive glycogen accumulation, partly polyglucosan	Extensive glycogen accumulation, partly polyglucosan	Extensive glycogen accumulation, partly polyglucosan
Muscle weakness	No	No	No
GYG1 genotype	c.304G > C; p.Asp102His (homo)	c.304G > C; p.Asp102His (homo)	c.248C > T; p.Thr83Met/c.487delG; p.Asp163Thrfs*5 (comp het)
Family history of cardiomyopathy	Negative	Negative	Negative
Cardiac interventions and treatment	ICD implantation. ACE inhibitor and β-blocker. Antiarrhythmic therapy (sotalol, later amiodarone) for a high PVC burden. Catheter ablation at age 44 y. Apixaban for paroxysmal atrial fibrillation	ICD with multiple interventions for recurrent arrhythmias. Guideline-directed medical therapy for heart failure	ICD and guideline-directed medical therapy for heart failure
Heart transplantation	No	Yes, at age 48 y	Yes, at age 36 y
Current cardiac status (most recent follow-up) at age years	Age 49 y: moderately dilated LV with EF 35%-40%, normal right ventricular function. Peak VO₂ 28 mL/kg/min (74% predicted). Clinically stable	Age 59 y: normal graft function, good exercise tolerance, no rejection episodes	Age 47 y: normal graft function, good exercise capacity, working full time
Noncardiac comorbidities	None reported	T2DM (after transplantation); overweight; hepatic steatosis	None reported

Abbreviations: ACE, angiotensin-converting enzyme; CMR, cardiac magnetic resonance imaging; comp het, compound heterozygous; EF, ejection fraction; GYG1, glycogenin-1 gene; homo, homozygous; ICD, implantable cardioverter-defibrillator; LGE, late gadolinium enhancement; LV, left ventricle; PVC, premature ventricular contraction; T2DM, type 2 diabetes mellitus; VO₂, oxygen uptake.

Patient 1 first presented at age 36 years, with recent onset of fatigue. Echocardiography showed normal cardiac dimensions and function with inferoposterior hypokinesia. Angiography was normal. Troponins were not elevated. Cardiac magnetic resonance (CMR) imaging was interpreted as acute lateral wall myocarditis.

At age 41 years, the patient presented acutely with sustained ventricular tachycardia and hemodynamic instability, requiring sedation and electrical cardioversion. CMR imaging revealed a mildly dilated left ventricle with slightly reduced ejection fraction (EF) and extensive fibrosis and fatty infiltration, predominantly in the lateral wall (Fig. 3A and 3B). The right chamber had normal size and function. High-sensitivity troponin T and N-terminal pro b-type natriuretic peptide (NT-proBNP) were mildly elevated (69 ng/L and 327 ng/L, respectively). An endomyocardial biopsy specimens showed, in a majority of the cardiomyocytes, large central regions with storage of PAS-positive material identical to what had been previously described in patients 2 and 3 (12, 13) (Fig. 4A). A skeletal muscle biopsy from the quadriceps of patient 1 showed a few scattered muscle fibers with polyglucosan storage (data not shown). Western blot of glycogenin-1 showed the presence of only nonglucosylated protein, indicating that the mutant glycogenin-1 was expressed but unable to autoglucosylate (Fig. 4B). He had no skeletal muscle weakness. Genetic testing identified a homozygous missense variant in the *GYG1* gene (c.304G>C, p.Asp102His, NM_004130.4). He received an implantable cardioverter defibrillator (ICD), and treatment with an angiotensin-converting enzyme inhibitor and a β-blocker was initiated.

**Figure 3:**
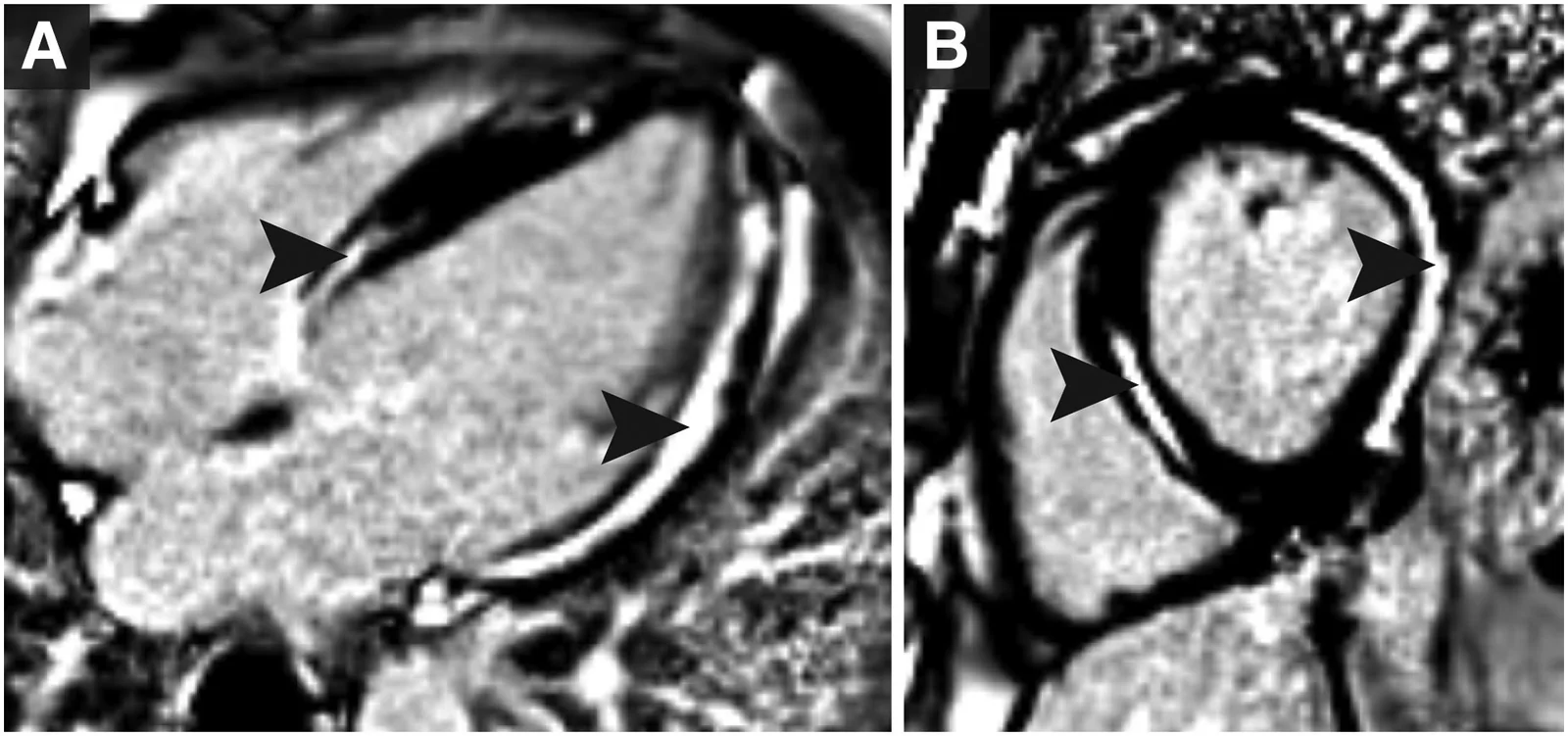
Cardiac magnetic resonance imaging (A, long axis; B, short axis) showing a slightly dilated left chamber and extensive increased signal (late gadolinium enhancement) in intramural and subepicardial layers, especially in the lateral wall and basal septum (arrows).

**Figure 4:**
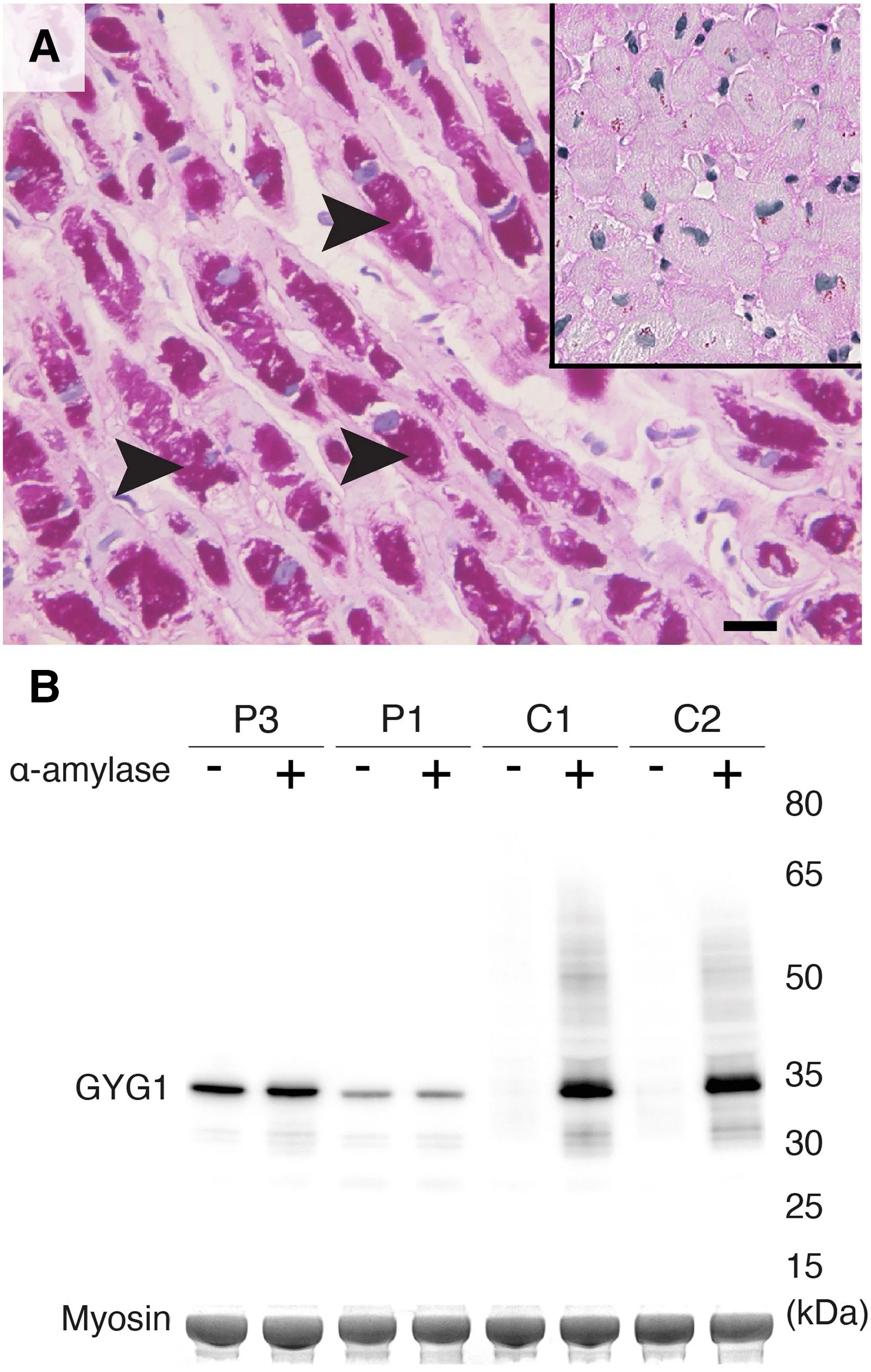
Morphological analyses of heart muscle in patient 1 and Western blot analysis of skeletal muscle in patients 1 and 3 and two controls. A, Endomyocardial biopsy showing accumulation of strongly PAS-positive material centrally located in most fibers (arrows) consistent with glycogen storage cardiomyopathy. Inset shows normal myocardium without glycogen accumulation. Paraffin-embedded sections stained with PAS. Bar corresponds to 25 µm. B, The Western blot analysis demonstrates that, in control muscle (C1 and C2), glycogenin-1 (GYG1) is identified only after α amylase digestion (+) since it is normally covalently bound to glycogen that will not enter the gel because of the large size of glycogen particles. In patients 1 and 3 (P1 and P3), α amylase digestion does not affect the result, demonstrating that all glycogenin-1 is free and thus not glucosylated and covalently bound to glycogen.

Over the next years the patient developed a highly symptomatic premature ventricular contraction (PVC) burden, initially treated with sotalol and later switched to amiodarone. Due to recurrence, catheter ablation was performed at age 46 with good effect. Amiodarone was weaned but had to be restarted due to recurrence of PVCs. Anticoagulation with apixaban was initiated for paroxysmal atrial fibrillation.

The patient has remained clinically stable with preserved exercise capacity. Cardiopulmonary exercise testing at age 48 years showed a peak workload of 172 W (74% predicted) and a peak oxygen uptake (VO₂) of 28 mL/kg/min. Echocardiography at age 49 years demonstrated a moderately dilated LV with an EF of 35% to 40%, normal right ventricular function, and an electrocardiogram showing incomplete right bundle branch block (QRS 98 ms) with inferolateral T-wave flattening. No muscle weakness has been observed.

Patient 2 is a 59-year-old man whose clinical history was described in detail up to the time of his cardiac transplantation at age 48 (patient 1 in (13)). He presented at age 36 with palpitations and exertional chest pain. Echocardiography showed severe cardiac hypertrophy and reduced EF (44%). Coronary angiography was normal. Myocardial biopsy showed extensive glycogen accumulation. He had no clinical evidence of skeletal muscle disease, but muscle biopsy showed occasional fibers with accumulation of glycogen inclusions. Over time, he developed worsening fatigue, progressive hypertrophy with ventricular dilation, and declining function (EF 28% by age 44). At age 45, CMR imaging revealed a severely dilated LV with marked dysfunction and extensive mid-myocardial/subepicardial scarring; coronary angiography was normal. He developed recurrent arrhythmias requiring ICD implantation, with multiple interventions. He ultimately underwent heart transplantation at age 48. He was homozygous for the same *GYG1* missense variant as patient 1, c.304G>C; p.Asp102His. The mutated glycogenin-1 protein was expressed, but unable to autoglucosylate (13). He is now 11 years post cardiac transplantation. He has normal cardiac function and normal 2- and 5-year coronary angiogram. He has good exercise tolerance on echocardiogram with all regions of the LV showing normal augmentation with appropriate wall motion. He has had no episodes of rejection. He is overweight and has type 2 diabetes, most likely related to immunosuppressive therapy. He has a large, echogenic liver that is likely a combination of steatosis and glycogen as per a liver biopsy at age 36 years. Liver ultrasound scan surveillance over 2 decades is unchanged. He has no clinical evidence of skeletal myopathy with no change in strength or power when last assessed by a neurologist at 57 years. Electromyogram/nerve conduction study at this time showed subtle changes in proximal muscles that could be consistent with myopathy, neurophysiological evidence of median neuropathy at the right wrist of moderate severity, consistent with carpal tunnel syndrome, and neurophysiological evidence of a mild length-dependent axonal sensory neuropathy, likely due to a combination of diabetes and tacrolimus treatment. He has no reported change in muscle strength.

Patient 3 was the first patient reported with a glycogenin-1–related disease in 2010 (12). He presented at age 27 years with a cardiac arrest due to ventricular fibrillation and was successfully resuscitated. On admission, the electrocardiogram showed LV hypertrophy and cardiac biomarkers were elevated. Coronary angiography revealed normal coronary arteries. Transthoracic echocardiography demonstrated a dilated LV with an EF of approximately 40%. CMR showed extensive late gadolinium enhancement involving the inferior and septal LV walls. Based on these findings, myocarditis was initially suspected. However, endomyocardial biopsy revealed marked glycogen accumulation with a partly fibrillar structure within cardiomyocytes (12). Skeletal muscle showed depletion of glycogen. Genetic investigations revealed that he was compound heterozygous for 2 variants in *GYG1*: c.248C>T; p.Thr83Met and c.487delG; p.Asp163Thrfs*5. The protein encoded from the allele with the missense variant was expressed but lacked the ability to autoglucosylate (12, 26, 27).

The patient received an ICD and was treated with guideline-directed medical therapy for heart failure. Despite optimal management, his cardiac function gradually deteriorated over the ensuing years, accompanied by progressive heart failure symptoms. At age 36 years, he underwent orthotopic heart transplantation.

Post transplantation, the patient has done well. He has not developed signs of increasing muscle weakness and a repeat skeletal muscle biopsy at age 40 showed glycogen depletion in about 50% of the fibers but no polyglucosan storage. At his most recent follow-up, at age 47 years, he demonstrated excellent graft function, good exercise capacity, and was working full time.

During the cardiomyopathy work-up, each patient was specifically asked about cardiac disease, sudden death, and muscle disease in first- and second-degree relatives. None of the patients reported a dysregulation of glycogen metabolism history of cardiac disease or of muscle symptoms compatible with GSD.

### Enriched proteins in the storage material in cardiomyocytes

Laser microdissection and liquid chromatography–tandem mass spectrometry cubed (LC*-*MS^3^) were performed on a specimen from patient 2 to identify proteins that were enriched in the storage material when compared to the surrounding cytoplasm of the cardiomyocytes. A list of proteins that were accumulated and the FC is presented in Supplementary Table S1 (22). In total, 217 proteins were identified and quantified in all 6 samples, including 3 different samples from the storage material and 3 different samples from the cytoplasm outside the storage material. A total of 47 proteins showed a significantly higher abundance in the storage material. Several of these enriched proteins were enzymes of glycogen metabolism (Fig. 5A-5D). Glycogen synthase 1 (*GYS1*) showed the highest FC of all proteins.

**Figure 5:**
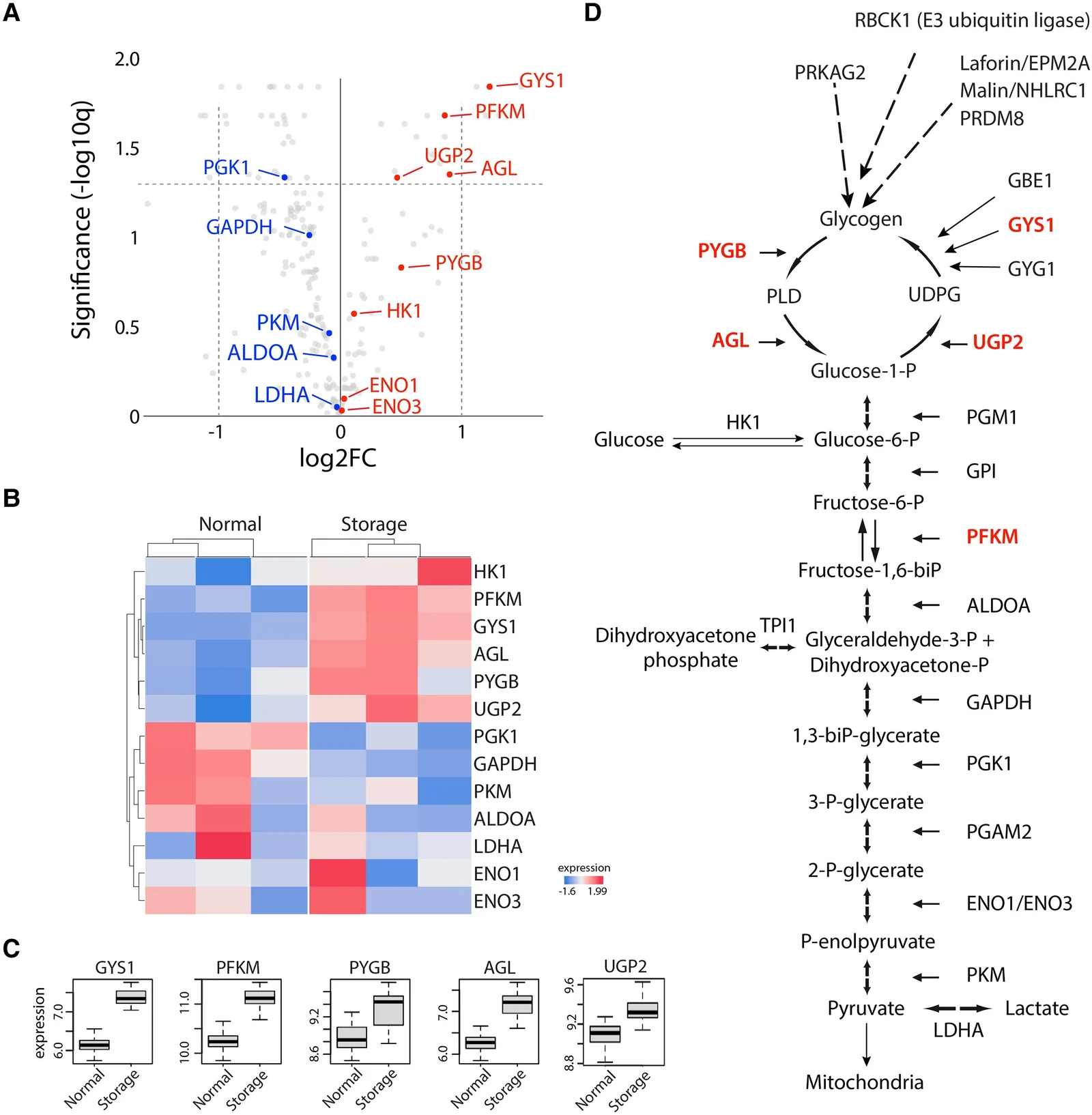
Proteomic characterization of differentially abundant proteins (DAP) in PAS-positive storage material with an emphasis on glycogen metabolism and glycolysis. Proteomic analysis was performed by comparing laser microdissected storage material (Storage) with adjacent cytoplasmic regions outside the storage material (Normal) from patient 2. A,Volcano plots depicting DAP associated with glycogen metabolism and glycolysis. B, Heat map of DAPs, color-coded according to high (red) or low (blue) abundance. Each column represents an individual sample. Dendrograms indicate hierarchical clustering. C, Box plots showing abundance of glycogen metabolism– and glycolysis-related proteins with the highest fold changes (log₂FC >0.4). D, Schematic overview of glycogen metabolism and glycolysis, highlighting (red) the most abundant proteins in the storage material.

IHC investigation was performed to validate the results from the proteomic investigation and also included other proteins that we previously found to be accumulated in the storage material in skeletal muscle tissue in PGBM1 (*GYG1*) and PGBM2 (*RBCK1*) (19, 20). For this IHC investigation, cardiac explants from 2 patients were included (patients 2 and 3) and compared with a donor heart explant. In line with the results based on LC-MS^3^, enriched proteins identified by IHC included glycogen synthase 1 (*GYS1*), debranching enzyme (*AGL*), glycogen phosphorylase (*PYGB*), UDP-glucose pyrophosphorylase 2 (*UGP2*), glycogenin-1 (*GYG1*), and laforin (*EPM2A*) (Fig. 6).

**Figure 6:**
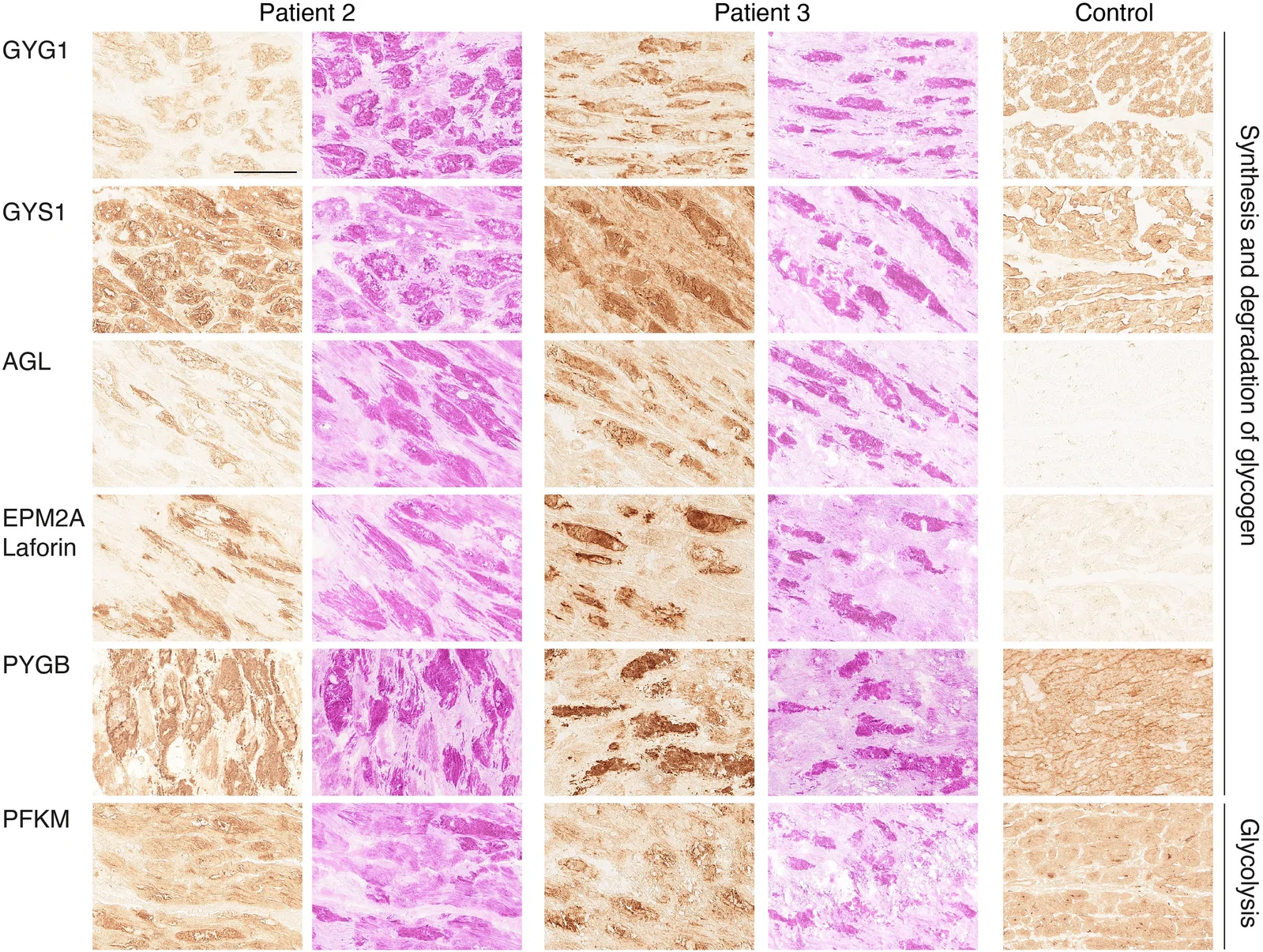
Immunohistochemical investigation of the PAS-positive storage material. Serial sections of cardiac muscle from patients 2 and 3 were analyzed using antibodies against selected proteins and periodic acid–Schiff (PAS) staining. The results show that proteins involved in glycogen metabolism are accumulated within PAS-positive storage material. AGL, glycogen debranching enzyme; EPM2A, laforin; GYG1, glycogenin-1; GYS1, glycogen synthase 1; PYGB, glycogen phosphorylase, brain type; PFKM, phosphofructokinase, brain type). The control represents normal myocardium. Bar corresponds to 100 μm.

The muscle-specific intermediate filament desmin (*DES*) and sequestosome 1 (*SQSTM1*, p62) were found to be enriched in the storage material in cardiomyocytes of the GSD15 patients (Fig. 7) and have previously been shown to be associated with polyglucosan storage in skeletal muscle fibers (19, 20). Sequestosome 1/p62 labels proteins for degradation by autophagocytosis or by proteasomes. Three other markers of protein degradation, including LC3, LAMP2, and ubiquitin, were not found to be enriched in a similar way (see Fig. 7). These 3 proteins were rather associated with lipofuscin that is normally present in cardiomyocytes (age pigment).

**Figure 7:**
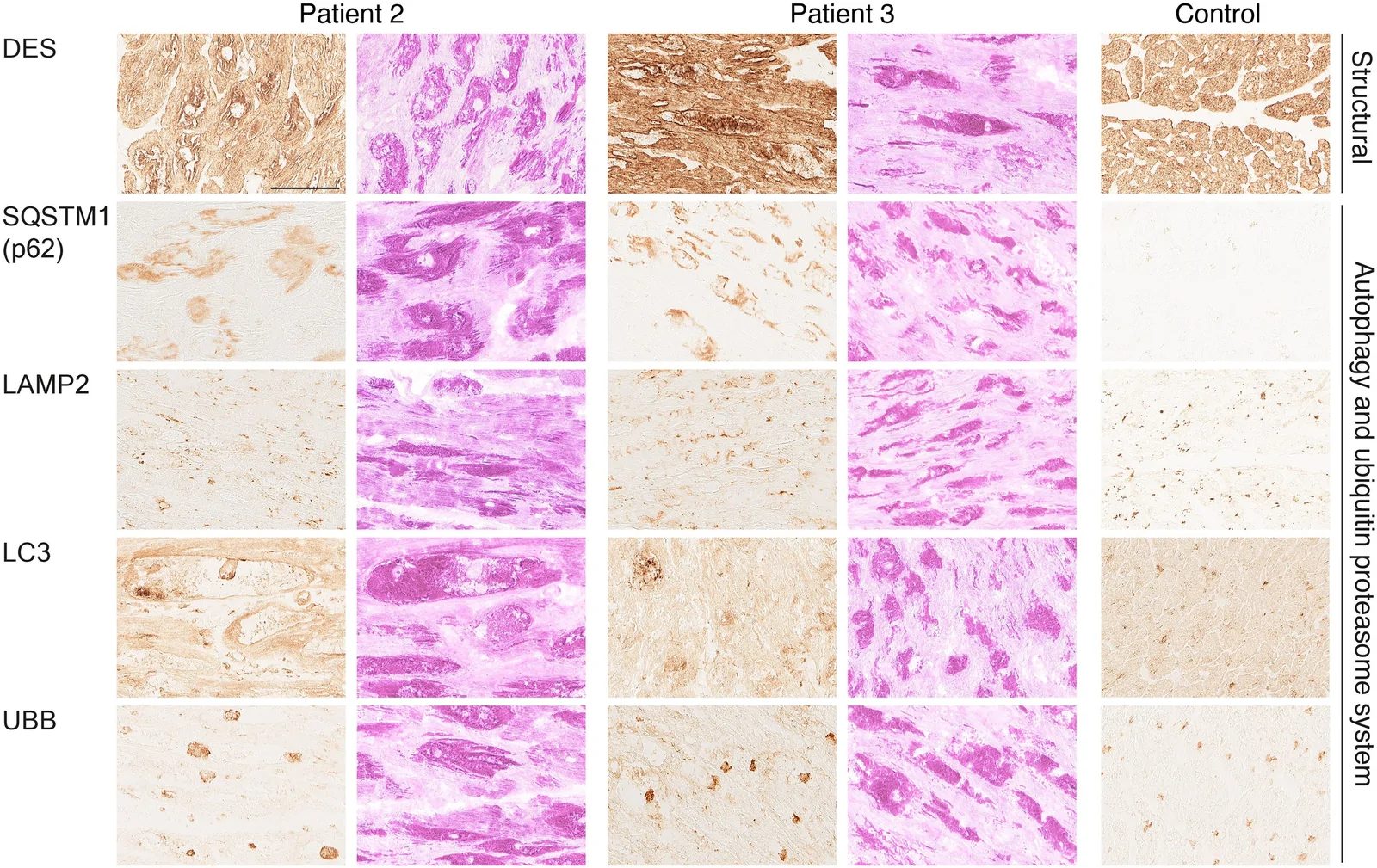
Immunohistochemical investigation of the PAS-positive storage material. Serial sections of cardiac muscle from patients 2 and 3 were analyzed using antibodies against selected proteins and periodic acid–Schiff (PAS) staining. The results show accumulation of desmin (DES) and sequestosome 1 (SQSTM1, p62) in PAS-positive storage material. Lysosome-associated membrane protein 2 (LAMP2); microtubule-associated protein 1, light chain 3, α (LC3), and ubiquitin B (UBB) do not show any clear accumulation corresponding to the PAS-positive storage material but rather punctuate deposition with a localization corresponding to the normal age pigment (lipofuscin). The control represents normal myocardium. Bar corresponds to 100 μm.

### Whole-tissue analysis by quantitative liquid chromatography–tandem mass spectrometry cubed

To obtain a global pattern of differently expressed proteins in glycogenin-1 associated cardiomyopathy, whole-tissue analysis of biopsy specimens from the LV of the explanted hearts was investigated by quantitative LC-MS^3^ in patients 2 and 3 compared to 4 apparently normal donor hearts. All biopsy specimens were labeled and analyzed in triplicate. In total, 4132 proteins were identified and quantified in all 18 samples. Upregulated or downregulated proteins with a log2FC greater than 0.5 or less than −0.5 and a false discovery rate less than 0.05 are shown in Supplementary Table S2 (22). Using these criteria, 241 proteins were found to be upregulated, and 159 proteins were downregulated in the glycogenin-1 cardiomyopathy hearts.

Globally upregulated proteins in the whole-tissue samples, which were also accumulated in the storage material (see Figs. 6 and 7), included PYGB, AGL, SQSTM1, and DES, whereas PFKM and GYS1 were globally downregulated. For GYS1, which was the most enriched protein in the storage material (see Fig. 5), this result indicates a redistribution of GYS1 from the cell cytoplasm to the PAS-positive storage material. GYG1, the mutated protein, was somewhat reduced in relation to controls with an FC of 0.3 and 0.4 in patients 2 and 3, respectively. This finding agrees with the Western blot results (see Fig. 4) and shows that in GSD15, glycogenin-1 is expressed and enriched in the storage material, as shown by IHC (see Fig. 6). The 10 and globally most upregulated proteins, with an FC greater than 3 (log2FC>1.6), are given in Fig. 8.

**Figure 8:**
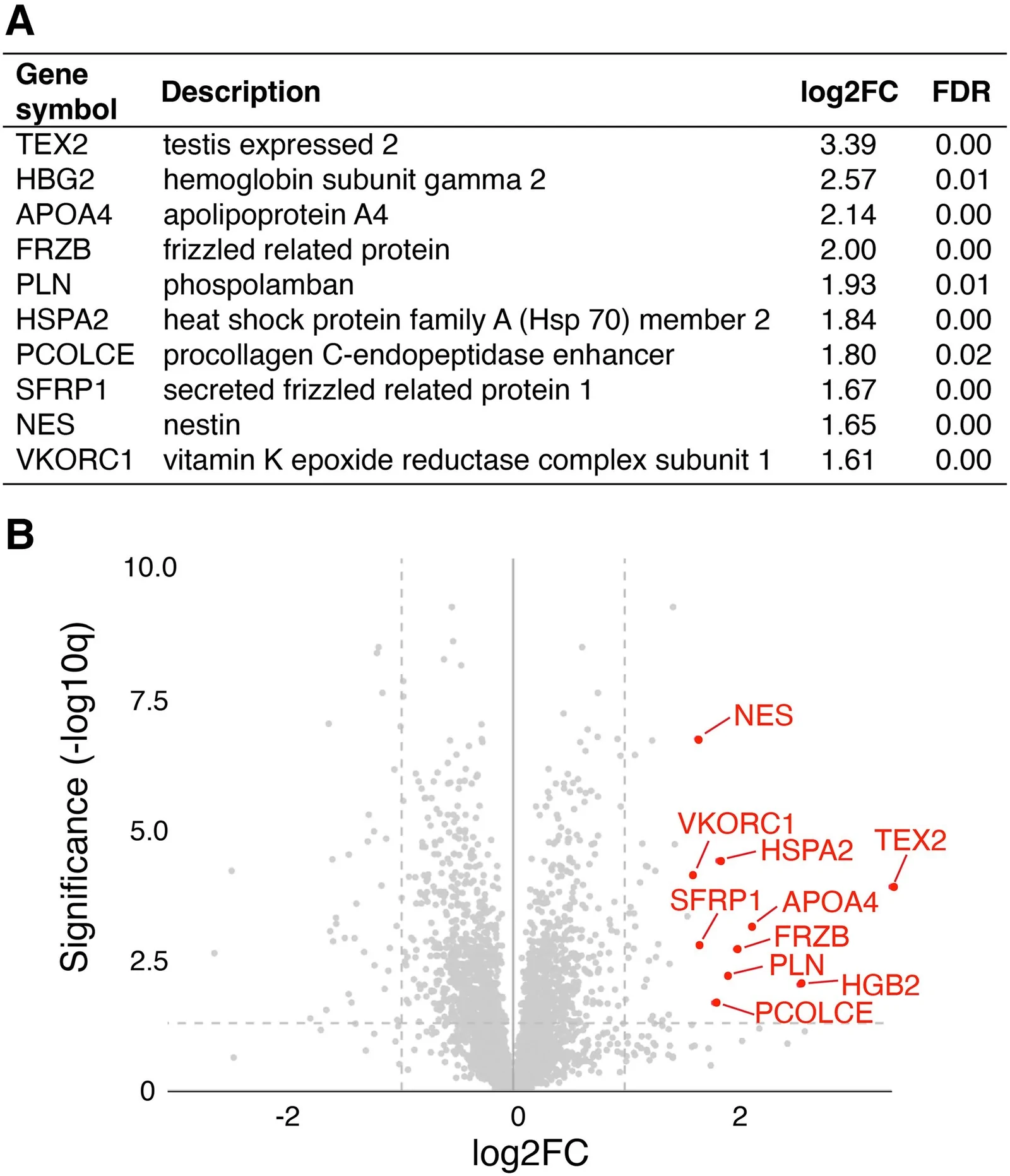
Upregulated proteins in whole tissue samples from explanted hearts. A, Table of the top 10 upregulated proteins. B, Volcano plot with the top 10 upregulated proteins marked in red.

The downregulated proteins showed, by STRING analysis, a clear clustering of mitochondrial proteins (Fig. 9, clusters 1 and 2). Analysis by enzyme- and IHC of the mitochondria revealed a reduced number of mitochondria in the large PAS-positive inclusions of the cardiomyocytes, whereas the surrounding cytoplasm of the cardiomyocytes appeared similar to the controls (Fig. 10). A corresponding lack of mitochondria in the PAS-positive storage material and normal-appearing mitochondria in the surrounding cytoplasm of the cardiomyocytes were apparent in electron micrographs (Fig. 11).

**Figure 9:**
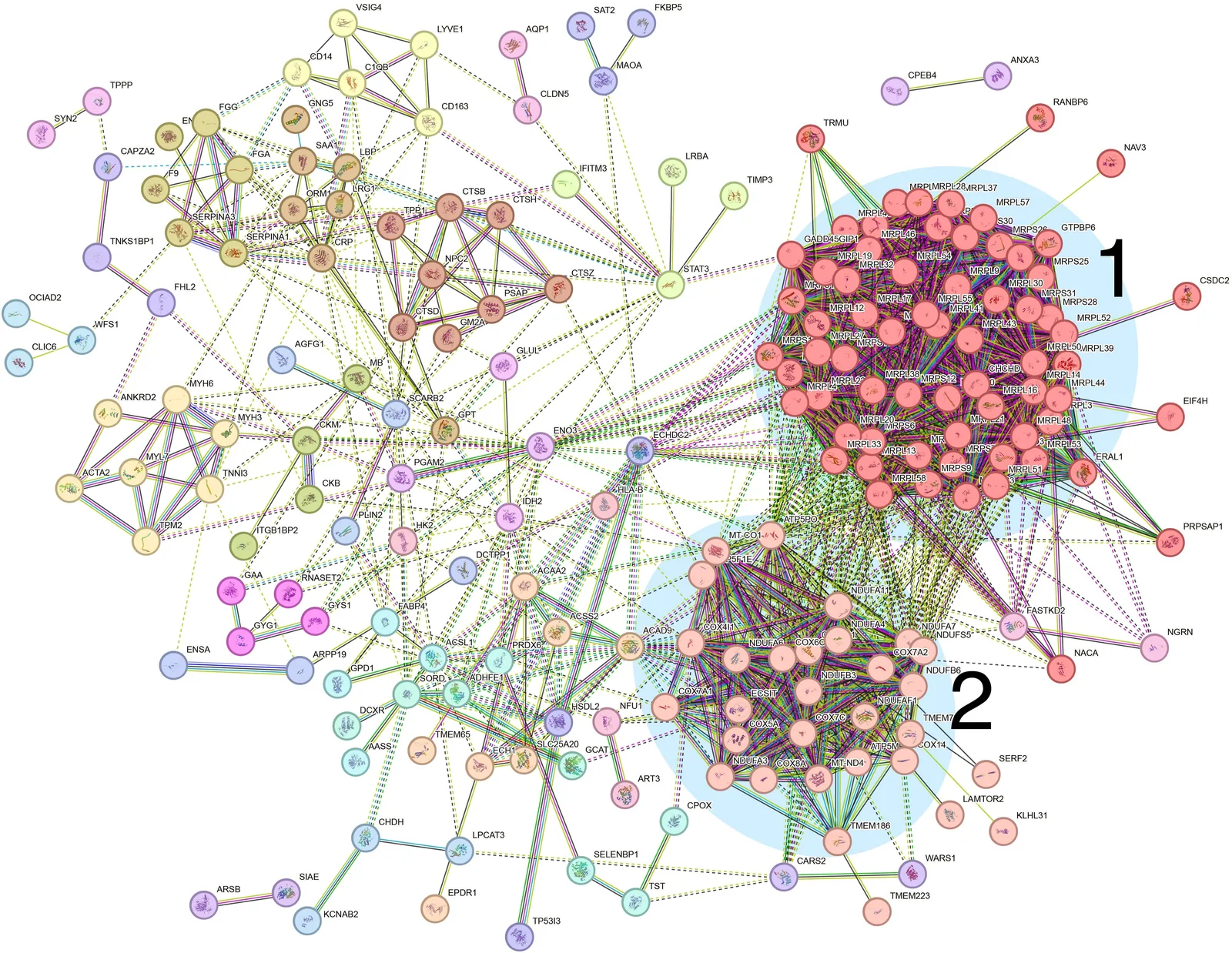
Proteomic analysis of low-abundance proteins from whole-tissue samples. From the STRING analysis, clusters were detected and 2 were highlighted: 1, proteins involved in mitochondrial translation; 2, proteins involved in the oxidative phosphorylation. (false discovery rate <0.05, log2FC >0.5, 217 proteins).

**Figure 10:**
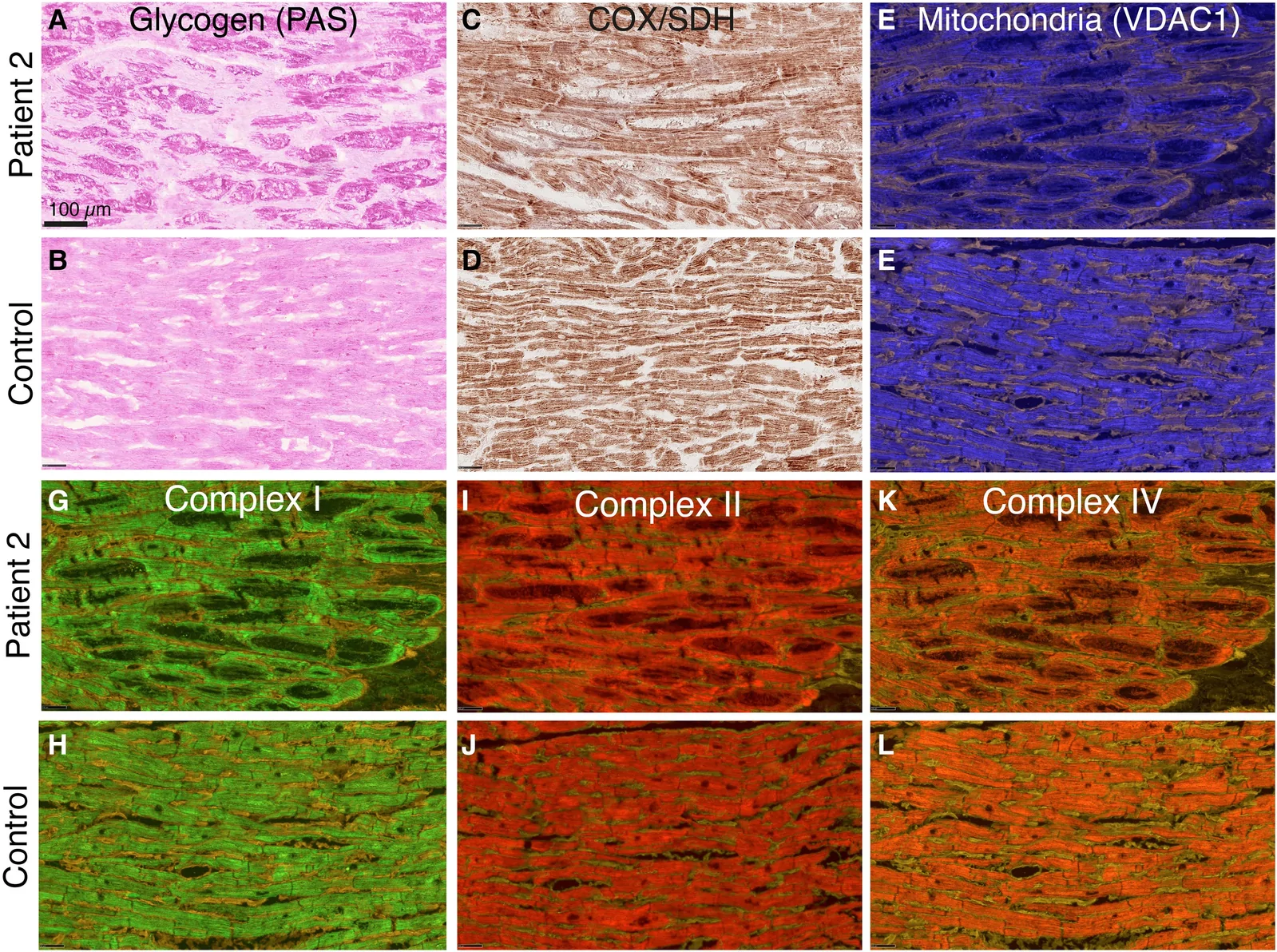
Distribution and functional analysis of mitochondria in the heart of a patient with glycogenin-1 cardiomyopathy and a control. A and B, PAS-positive storage material is present in the majority of the cardiomyocytes in the patient but absent in controls. C to L, By enzyme histochemistry of cytochrome c oxidase, combined with succinate dehydrogenase (COX/SDH), and immunofluorescence of the mitochondrial membrane protein VDAC1 and complex I, II, and IV of the respiratory chain, the regions with PAS-positive storage of material in the cardiomyocytes in the patient appear as empty vacuoles in the center of each cell. This may cause an overall reduction of mitochondria in the tissue. The amount and activity of mitochondria outside the storage material is comparable to that of the control.

**Figure 11:**
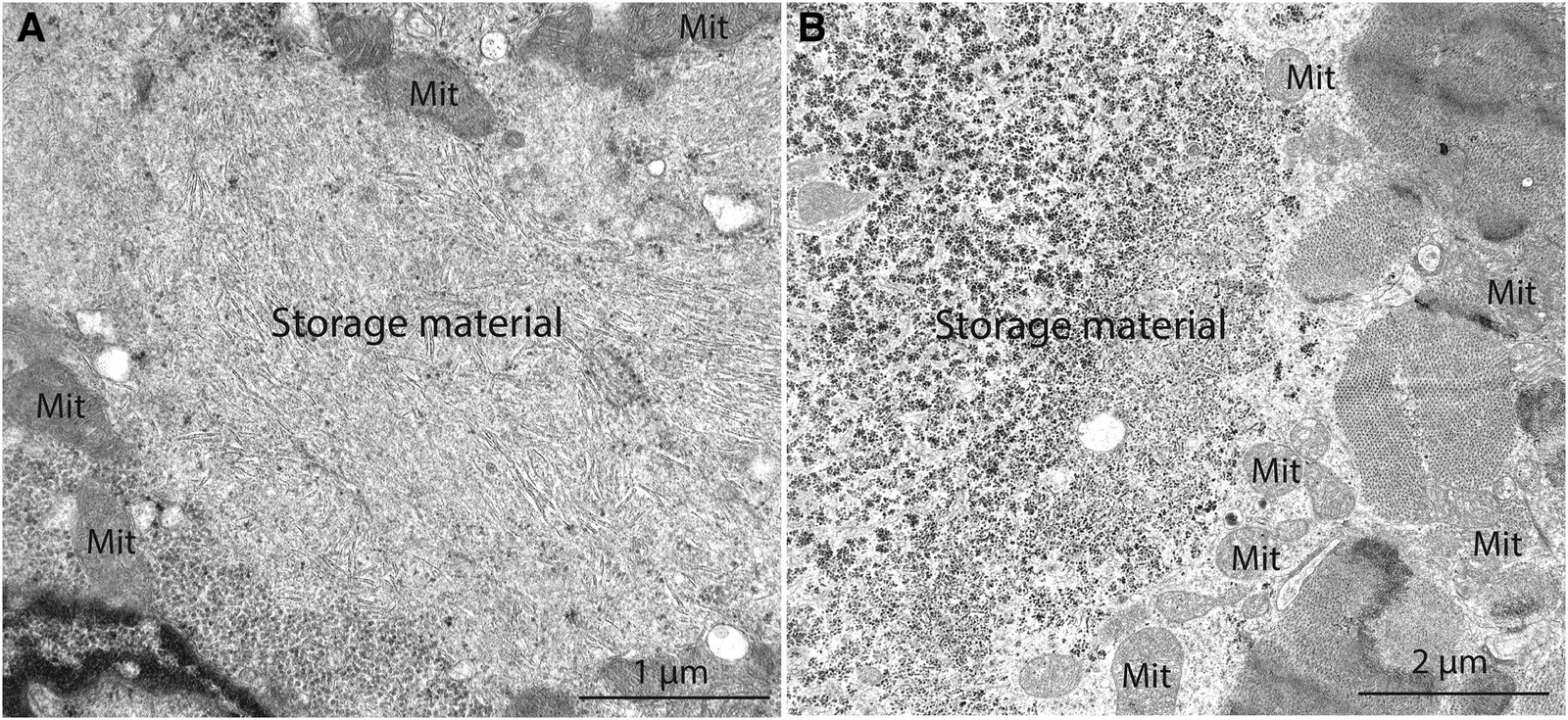
Electron micrographs of storage material in the cardiomyocytes. A and B, The storage material has a partly fibrillar structure as seen in A, but in other regions, B, it is granular but mixed with fibrillar structures. Few mitochondria are seen in the storage material, but rather associated with the myofibrils in the cytoplasmic regions surrounding the storage material. Mit, mitochondria.

## Discussion

In this study, we report the results of combined proteomic and immunohistochemical analyses of storage material in cardiomyocytes from patients with GSD15, a form of cardiomyopathy caused by the expression of mutated glycogenin-1 lacking normal autoglucosylation properties. To date, including the present report, 5 unrelated patients have been described in the literature (12, 13). All patients exhibited a characteristic accumulation of glycogen in large, circumscribed regions within cardiomyocytes, partly in the form of polyglucosan, which has a fibrillar ultrastructure.

Our results show that, in the PAS-positive storage material, there is accumulation of proteins involved in cardiac glycogen metabolism, including UDP-glucose pyrophosphorylase 2 (UGP2), glycogenin-1 (GYG1), glycogen synthase (GYS1), laforin (EPM2A), glycogen phosphorylase B (PYGB), and debranching enzyme (AGL). Importantly, a distinction can be made between proteins that are globally upregulated, as identified by whole-tissue proteomic analysis, such as PYGB (FC 3.0) and AGL (FC 2.7), and proteins that are redistributed to the inclusions without evidence of overall upregulation, including UGP2, GYG1, GYS1, and laforin. These findings suggest that enzymes involved in glycogen synthesis are redistributed to the PAS-positive material rather than generally upregulated, whereas enzymes involved in glycogen degradation are both redistributed and globally increased.

Glycogen synthase 1 is a key enzyme in glycogen synthesis, and although an overall downregulation was shown by whole-tissue analysis from the GSD15 patients (FC 0.7), it was among the most enriched proteins within the storage material (FC 2.3). Glycogen synthase plays a central role in the formation of polyglucosan, as demonstrated in branching enzyme deficiency, where its relative overactivity compared with the branching enzyme leads to the production of elongated, poorly branched and amylopectin-like glucan chains that are less soluble than normal glycogen (28, 29). Accordingly, a direct association between glycogen synthase accumulation and polyglucosan deposition in GSD15 appears likely. This concept is further supported by findings from animal models of polyglucosan storage diseases, including Lafora disease (*EPM2A*), adult polyglucosan body disease (*GBE1*), and *RBCK1*-associated cardiomyopathy and myopathy, in which inactivation or downregulation of glycogen synthase ameliorates disease manifestations (30, 31). These observations indicate that dysregulation of glycogen metabolism involving glycogen synthase is a key element in polyglucosan formation and pathogenically important in GSD15. Polyglucosan is insoluble and resistant to degradation by glycogenolysis, rendering the myocardium energetically compromised under stress despite its glycogen-rich appearance. In addition, polyglucosan aggregates disrupt sarcomeric and mitochondrial architecture and promote chronic myocyte injury with subsequent reactive fibrosis. These processes contribute to progressive restrictive physiology and, ultimately, systolic dysfunction, as observed in cardiomyopathies associated with polyglucosan storage (13, 32-35).

The mechanism underlying the redistribution and accumulation of glycogen synthase in cardiomyocytes in GSD15 remains unclear. However, our data show that the mutant glycogenin-1 itself accumulates within the storage material. Glycogenin-1 forms together with glycogen synthase through specific binding sites a complex that is essential for the function and regulation of glycogen synthase (36-39). Through its interaction with glycogen synthase, mutant glycogenin-1 may therefore contribute to the aberrant accumulation and dysregulation of glycogen synthase and, consequently, to polyglucosan accumulation in the heart.

Protein accumulation within PAS-positive inclusions included not only proteins associated with glycogen metabolism but also others, such as the intermediate filament desmin; however, electron microscopy revealed no formation of classic protein aggregates, as in desmin cardiomyopathy (18). The marked global upregulation (FC 2.3) and accumulation of sequestosome 1 (p62) within the storage material indicate that some of the accumulated proteins in the storage material are tagged for proteasomal or lysosomal degradation. However, we observed no increase in the autophagosome marker LC3, the lysosomal marker LAMP2, or ubiquitin. These findings suggest that p62-labeled proteins do not enter the autophagic or proteasomal degradation pathways but instead remain sequestered within the inclusions. The pattern of sequestosome 1 (p62) immunostaining is also different from what is seen in most protein aggregate myopathies. In GSD15 and other polyglucosan storage diseases, the sequestosome 1 (p62) immunoreactive material is more widespread within the storage material than in protein aggregate myopathies, where it is frequently seen as smaller aggregates associated with autophagic vacuoles (18).

Quantitative LC-MS^3^ analysis of whole myocardial tissue was performed in 2 patients who had undergone cardiac transplantation, enabling comparison between protein levels in whole-tissue analyses and proteins specifically accumulated in the storage material, as well as identification of globally dysregulated proteins. The 10 most upregulated proteins all showed FCs greater than 3 (see Fig. 8). Five of these proteins (FRZB, HSPA2, PCOLCE, SFRP1, and NES) are established markers of cardiac disease, including heart failure of various etiologies, and are likely nonspecific (40-44). Notably, nestin, typically expressed in early fetal cardiomyocytes, has been associated with myocardial regeneration under certain conditions (45, 46). The exact roles of these and several other globally upregulated proteins in GSD15 remain to be studied.

The globally downregulated proteins in the myocardium of the 2 GSD15 patients were mainly associated with mitochondrial function as revealed by STRING analysis. To further explore the involvement of the respiratory chain and mitochondrial distribution, we performed enzyme histochemical, immunofluorescence, and electron microscopical analyses. An apparent explanation for the reduction in mitochondrial proteins was the lack of mitochondria in the large inclusions of PAS-positive material, rather than impaired function of individual mitochondria. However, our data indicate that there was an overall reduction of mitochondria, which possibly contributed to the cardiac failure. Impaired mitochondrial function has been implicated in cardiac failure of various etiologies, and in GSD15, the role of mitochondrial deficiency needs to be further investigated. However, in all patients a clinical deterioration appeared in adulthood and was concomitant with extensive signs of myocardial fibrosis as revealed by late gadolinium uptake on CMR and also seen in the cardiac explants at transplantation.

In addition to the proteomic investigation of storage material in GSD15, we have, in this study, reported the clinical follow-up of the 2 patients reported with GSD15 (12, 13) and a previously not reported patient. This information adds to a previous report on 3 patients with GSD15 and cardiomyopathy (13). All show a similar pattern of marked storage of PAS-positive material in cardiomyocytes and myocardial damage and fibrosis, and progressive heart failure. Of special interest is the comparison with polyglucosan body myopathy type 2 (PGBM2), a pure skeletal muscle disease without signs or symptoms of cardiomyopathy but caused by recessive pathogenic variants in the same gene, *GYG1*. In GSD15 with cardiomyopathy, there is expression of mutated glycogenin-1, with loss of normal autoglucosylation properties, as demonstrated by IHC, Western blot analysis, and MS. On the other hand, PGBM2 is usually caused by loss-of-function variants, and Western blot reveals the absence or near-complete lack of glycogenin-1 expression. It therefore appears that lack of glycogenin-1 expression is less harmful to the heart than the expression of a dysfunctional protein. In this respect, cardiac and skeletal muscle seem to be different, but the reason for this difference is not yet understood. In this context, it is of interest to notice that glyogenin-1 knockout mice do not show any similar storage disease in muscle or heart, although there is an overall accumulation of glycogen in muscle tissue (47).

The accumulated storage material is partly typical of polyglucosan, showing a fibrillar structure and being resistant to α amylase digestion, but most of the storage material seems to be less resistant to amylase digestion as compared with what is seen in *RBCK1*-associated polyglucosan storage cardiomyopathy and skeletal myopathy (PGBM1) (34).

It is also worth noting that 4 of 5 published cases of GSD15 are homozygous for the p.Asp102His *GYG1* missense variant (Supplementary Table S3 (22)). This may partly be explained by the fact that this allele is less rare than other pathogenic *GYG1* missense variants, such as c.248C>T; p.Thr83Met in patient 3 (see Supplementary Table S3 (22)). GSD15 is apparently a rare disease but probably underdiagnosed, since the clue to diagnosis requires endomyocardial biopsy and PAS staining, or genetic testing, including the *GYG1* gene, which is not performed in all cardiomyopathy patients. With the increasing use of genetic testing, more cases will probably be identified, and the phenotypic spectrum will be clarified.

Storage cardiomyopathies such as Fabry disease, Danon disease, and the GSDs, and infiltrative cardiomyopathies such as amyloidosis, all phenocopy hypertrophic cardiomyopathy and share a restrictive physiology with preserved EF in the early stages, progressing to systolic dysfunction and chamber dilation later. For several of these conditions, disease-modifying treatment is now available, including enzyme replacement and chaperone therapy in Fabry disease (48) and tafamidis and gene-silencing agents in transthyretin amyloidosis (49). No such specific treatment exists for GSD15, and management remains symptomatic, with guideline-directed heart failure therapy, device therapy where indicated, and cardiac transplantation in end-stage disease.

Although no disease-modifying treatment currently exists for GSD15, the pathobiology points to several conceptual therapeutic directions. Substrate reduction through pharmacological inhibition of glycogen synthase could in principle limit polyglucosan accumulation (30, 31). Gene therapy or enzyme replacement therapy to restore functional glycogenin-1 is a theoretical option, although the scaffold function of glycogenin-1 makes enzyme replacement less straightforward than in Pompe disease (50). Enhancement of autophagic clearance has also been proposed for polyglucosan disorders (51). None of these approaches is yet clinically available. Glycogenin-1 is considered essential for glycogen metabolism because it provides the necessary primer for de novo glycogen synthesis. However, there appear to be no clinical signs of cardiomyopathy or general metabolic dysfunction in GSD15 until adulthood, when degenerative features with myocardial fibrosis become apparent. Interventions aiming at modulating the metabolic function of the epicardial adipose tissue are an emerging research field in cardiometabolic heart disease (52), but whether it will have a role in the treatment of GSD15 remains to be investigated.

In conclusion, we have reported a new case of GSD15 and investigated the protein content of the storage material in cardiomyocytes that is characteristic of this disease. The results support the concept that GSD15 is a distinct entity different from PGBM2, and that accumulation of glycogen synthase and other enzymes involved in glycogen metabolism, in addition to the presence of mutated glycogenin-1, is involved in the dysregulation of glycogen synthesis and the pathobiology of GSD15 cardiomyopathy.

## Data Availability

The datasets generated during the current study are not publicly available but are available from the corresponding author on reasonable request.

## Abbreviations

CMR: cardiac magnetic resonance
EF: ejection fraction
GSDs: glycogen storage diseases
GSD15: glycogen storage disease type 15
*GYS1*: glycogen synthase 1
ICD: implantable cardioverter-defibrillator
IHC: immunohistochemistry
LCM: laser capture microdissection
LC-MS^3^: liquid chromatography–tandem mass spectrometry cubed
log2FC: log2 fold change
LV: left ventricle
MS: mass spectrometry
p62: sequestosome 1
PAS: periodic acid–Schiff
PGBM2: polyglucosan body myopathy type 2
PVC: premature ventricular contraction
TMT: tandem mass tags
VO₂: oxygen uptake
